# Human dCTP pyrophosphatase 1 promotes breast cancer cell growth and stemness through the modulation on 5-methyl-dCTP metabolism and global hypomethylation

**DOI:** 10.1038/oncsis.2015.10

**Published:** 2015-06-15

**Authors:** F-f Song, L-l Xia, P Ji, Y-b Tang, Z-m Huang, L Zhu, J Zhang, J-q Wang, G-p Zhao, H-l Ge, Y Zhang, Y Wang

**Affiliations:** 1 Department of Immunology and Microbiology, Shanghai Institute of Immunology, Shanghai Jiaotong University School of Medicine, Shanghai, China; 2 Shanghai-MOST Key Laboratory of Health and Disease Genomics, Chinese National Human Genome Center at Shanghai, Shanghai, China; 3 Department of Pharmacology, Shanghai Jiaotong University School of Medicine, Shanghai, China; 4 Department of Microbiology and Microbial Engineering, School of Life Sciences, Fudan University, Shanghai, China

## Abstract

Human DCTPP1 (dCTP pyrophosphatase 1), also known as XTP3-transactivated protein A, belongs to MazG-like nucleoside triphosphate pyrophosphatase (NTP-PPase) superfamily. Being a newly identified pyrophosphatase, its relevance to tumorigenesis and the mechanisms are not well investigated. In the present study, we have confirmed our previous study that DCTPP1 was significantly hyperexpressed in breast cancer and further demonstrated its strong association with tumor progression and poor prognosis in breast cancer. Knockdown of *DCTPP1* in breast cancer cell line MCF-7 cells remarkably retarded proliferation and colony formation *in vitro*. The capacity of mammosphere formation of MCF-7 was suppressed with the silence of *DCTPP1*, which was consistent with the enhanced mammosphere-forming ability in *DCTPP1*-overexpressed MDA-MB-231 cells. To further dissect the mechanisms of DCTPP1 in promoting tumor cell growth and stemness maintenance, its biochemical properties and biological functions were investigated. DCTPP1 displayed bioactive form with tetrameric structure similar to other MazG domain-containing pyrophosphatases based on structure simulation. A substrate preference for dCTP and its methylated or halogen-modified derivatives over the other canonical (deoxy-) NTPs was demonstrated from enzymatic assay. This substrate preference was also proved in breast cancer cells that the intracellular 5-methyl-dCTP level increased in *DCTPP1*-deficient MCF-7 cells but decreased in *DCTPP1*-overexpressed MDA-MB-231 cells. Moreover, global methylation level was elevated in *DCTPP1*-knockdown MCF-7 cells or mammosphere-forming MCF-7 cells but decreased significantly in *DCTPP1*-overexpressed MDA-MB-231 cells and its mammospheres. Our results thus indicated that human DCTPP1 was capable of modulating the concentration of intracellular 5-methyl-dCTP. This in turn affected global methylation, contributing to a known phenomenon of hypomethylation related to the cancer cell growth and stemness maintenance. Our current investigations point to the pathological functions of DCTPP1 overexpression in breast cancer cells with aberrant dCTP metabolism and epigenetic modification.

## Introduction

Intrinsic physiological processes such as cell metabolism or extrinsic oxidative damage and pathogen infection^1, 2^ are prone to generate noncanonical nucleotides. Incorporation of noncanonical nucleotides into DNA probably leads to increased mutagenesis and DNA damage, which is detrimental to genomic stability and integrity.^3^ One of the preventive mechanisms to maintain the purity of nucleotide pools in cells is to develop elaborate enzymatic repairing systems,^4, 5^ among which is a class of enzymes named nucleoside triphosphate pyrophosphatases (NTP-PPases).^6^ NTP-PPases function by hydrolyzing the α–β phosphodiester bond of (d)NTPs to produce corresponding monophosphate and PPi. All the NTP-PPases identified can be classified into four superfamilies: dUTPase, ITPase, Nudix (nucleoside diphosphate linked to an X moiety, or MutT-like) hydrolase and all-α NTP-PPase (MazG).^6^ The properties and functions of certain typical NTP-PPase molecules have been well described. dUTPase, which binds to dUTP with high affinity and shows little catalytic activity against other NTPs, is a key molecule to control dUTP concentration in intracellular nucleotide pool.^7^ dUTPase exists extensively from prokaryote to eukaryocyte organisms. In *Escherichia coli* (*E. coli*), mutations in the *dut* gene are either lethal or increase the incorporation of uracil into genomic DNA.^8^ dUTPase activity is also essential for survival in yeast.^9^ ITPase is firstly described in human erythrocyte.^10^ All characterized ITPases hydrolyze ITP and XTP, whereas do not recognize canonical NTPs. Its homologs in other organisms show the capacity of protecting hosts from the mutagenic effects of the base analog HAP.^11^ MutT from *E. coli* is the best characterized Nudix hydrolase specifically catalyzing the oxidative noncanonical nucleotides, such as 8-oxo-GTP.^12, 13^ Deletion of *mutT* in *E. coli* results in the increased AT to CG mutation up to 100–3200 folds.^13^ In human, MTH1, a MutT-homology enzyme, is also catalytic against 8-oxo-dGTP as well as another two oxidized dNTPs, 2-oxo-dATP and 8-oxo-dATP,^14^ indicating its house-cleaning property. MazG is a typical member of all-α NTPase superfamily. MazG from *Mycobacterium tuberculosis* (*Mtb*) is demonstrated to respond to oxidative stress by catalyzing oxidized noncanonical nucleotides with specificity to 5-OH-dCTP and prevent them from incorporating into DNA or RNA.^15^ Direct incorporation of 5-OH-dCTP into *mazG*-null mutant strain of *Mycobacterium smegmatis* (*Msm*) leads to a dose-dependent CG-TA transition.^15^ Therefore, NTP-PPases join hands with DNA repair enzymes to guarantee the genetic stability through degrading the noncanonical nucleotides to maintain normal life cycle of cells.^16^

Moreover, NTP-PPases are also described to be associated with carcinogenesis. For example, the depletion of *mth1* in mice leads to a higher incidence of spontaneous tumorigenesis.^17^ In human MTH1 has been reported to be overexpressed in multiple cancers and is involved in the resistance to oxidization stress.^18, 19^ Overexpression of MTH1 can prevent H-RAS-induced DNA damage response and premature senescence, whereas the loss of MTH1 preferentially induces an *in vitro* proliferation defect in RAS-transformed tumorigenic cells.^20^ dUTPase is another NTP-PPase dedicated to carcinogenesis. Studies reveal that dUTPase is significantly overexpressed in hepatocellular carcinomas. Its expression level is strongly correlated with histological grades and a poor prognosis.^21^ There is also evidence of elevated expression of dUTPase in NSCLC cell lines and fresh tumor specimens.^22^ However, the expression of dUTPase in colon cancer is highly variable in quantity and diverse in intracellular localization.^23^ Noteworthy, the expression of nucleic dUTPase isoform in normal cells is proliferation associated while dUTPase is mostly expressed in replicating cells of normal tissues.^23^ All the above studies indicate that human NTP-PPases might have an important role in cancer progression and prognosis to some extent.

Using MazG sequence as query and searching by iterative PSI-BLAST, we newly defined a MazG ortholog in human named dCTP pyrophosphatase 1 (DCTPP1), which contains a MazG domain with high similarity to bacteria MazG sequences.^24^ DCTPP1, also referred to as XTP3-transactivated protein A, is the first dCTP pyrophosphatase identified in human whose function has been poorly investigated. Transcriptional microarray profiling and SAGE data in the NCBI's GEO database suggest that DCTPP1 is highly expressed in embryonic and proliferating cells, including the liver, kidney, ovary and testis.^25, 26, 27^ Our previous work showed that DCTPP1 was prevalently expressed in multiple carcinomas and intended to accumulate in the nucleus of cancer cells in multiple tumors,^28^ which suggests the potential role of DCTPP1 in cancer progression. Recently, Requena *et al.*^29^ has introduced its biochemical properties and suggested its role in the preservation of genome integrity. Morisaki T *et al.*^30^ revealed its association with the development and prognosis of gastric cancer. However, its involvement in tumorigenesis is not clearly addressed. In the present study, we have precisely defined its properties and pathological significance of DCTPP1. Our findings demonstrated that human DCTPP1 was involved in promoting breast cancer cell proliferation and stemness maintenance, largely through controlling 5-methyl-dCTP metabolism and global DNA hypomethylation. Our study thus provides the evidence on how the DCTPP1-mediated nucleotide metabolic machinery is being engaged in tumorigenesis.

## Results

### DCTPP1 is highly expressed in breast tumor tissues and significantly associated with the poor overall survival and prognosis in breast cancer

**Figure 1:**
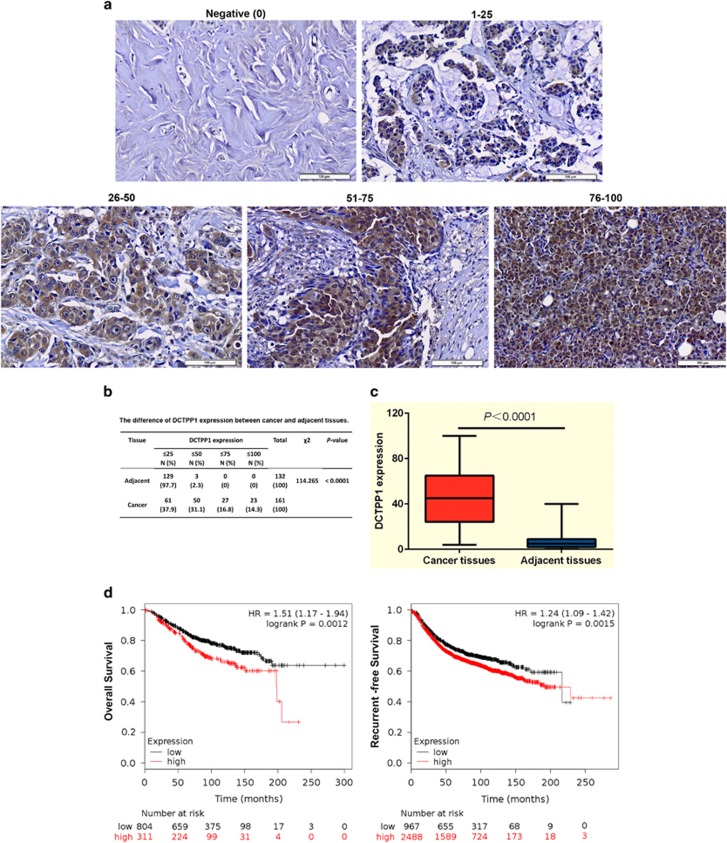
DCTPP1 expression in breast cancer tissues and its correlation with overall survival and prognosis of breast cancer patients. (**a**) Immunohistochemical staining was performed on breast cancer tissue microarray (TMA) for the detection of DCTPP1. DCTPP1 expression scores of adjacent and tumor tissues were indicated on each figure. (Scale bar, 100  μm). (**b**) Samples in breast TMA were subgrouped based on the percentage of DCTPP1-positive cells and the DCTPP1 expressing intensity. The difference in adjacent tissues (*n*=131) and breast cancer tissues (*n*=161) was analyzed by *χ*^2^-test. (**c**) Statistic analysis of DCTPP1 expression scores in breast cancer and adjacent tissues was performed by GraphPad Prism 6.0 (GraphPad Software, Inc., San Diego, CA, USA). DCTPP1 expression in breast cancer tissues was markedly higher than that in paired adjacent tissues (*P*<0.0001). (**d**) Overall survival and the cumulative recurrence-free survival curves of patients with high or low expression of DCTPP1 in breast cancer tissues were evaluated by Kaplan–Meier curves.

On the basis of our previous work,^28^ we further verified the expression profiling of DCTPP1 in breast cancer here. By using tissue microarrays, 161 breast cancer tissues and 132 paired adjacent tissues were conducted for the determination of DCTPP1 expression. It was found that DCTPP1 was significantly overexpressed in cancerous tissues when compared with the adjacent regions (*P*<0.0001) (Figures 1b and c). To compare quantitatively, only 2.3% (3 in 132 cases) of matched adjacent tissues scored >25. Breast cancerous tissues (62.2% (100 in 161 cases)) under investigation exhibited DCTPP1 expression with a score exceeding 25 (Figures 1b and c). It was notable that although DCTPP1 was detectable in both the nucleus and cytoplasm in cancerous tissues, positive signals of DCTPP1 were mainly localized in the nucleus of tumor cells (Figure 1a).

Correlation between DCTPP1 expression and clinicopathological parameters was further analyzed in 161 cases of breast cancers. Higher DCTPP1 expression was related to higher tumor stage (*P*=0.001) and tumor grade (*P*=0.018). But no evident correlations were observed between DCTPP1 expression and other clinical features such as histological classification and ER, PR or HER-2 expression (Table 1). We also performed the correlation study between *DCTPP1* mRNA level and disease prognosis using the Kaplan–Meier plotter web tool.^25^ The results revealed that patients with high DCTPP1 expression (311 cases) showed lower probability of overall survival compared with those with low DCTPP1 expression (804 cases; *P*=0.0012, Figure 1f). Moreover, patients in the high-expression group (2488 cases) exhibited a poorer prognosis indicated by recurrence-free survival probability when compared with 967 patients in the low-expression group (*P*=0.0015, Figure 1g). These results indicate that DCTPP1 is strongly associated with breast cancer progression and might be indicative to evaluate the prognosis in breast cancer.

### Knockdown of *DCTPP1* retards cell proliferation of MCF-7 cells *in vitro*

**Figure 2:**
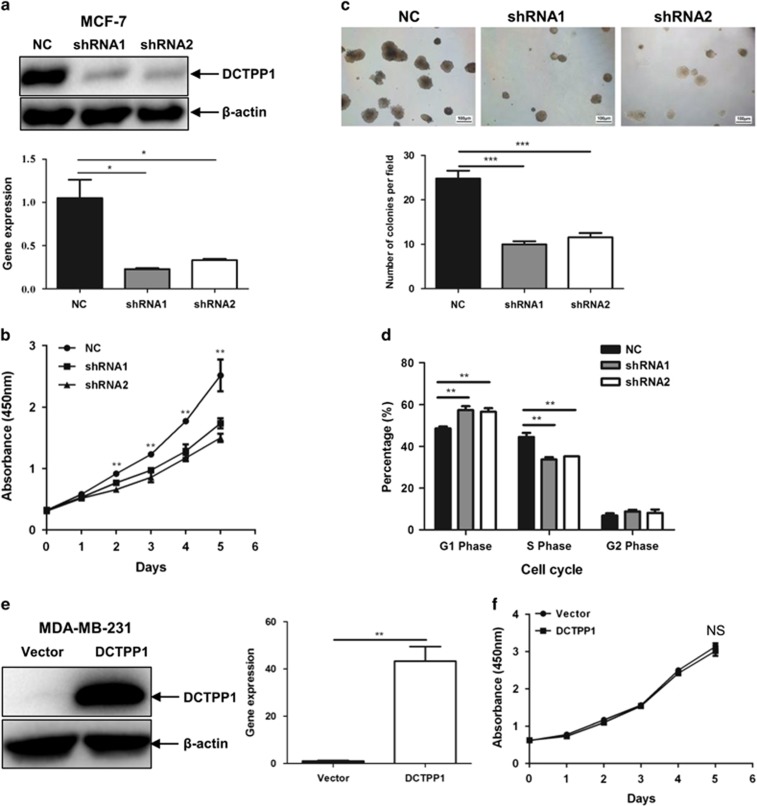
Suppression of DCTPP1 markedly inhibits the proliferation of MCF-7 *in vitro*. (**a**) The suppression of DCTPP1 expression in *DCTPP1*-knockdown MCF-7 cells was determined by western blotting (upper panel) and real-time quantitative PCR with β-actin as internal reference (lower panel). (**b**) Proliferative assay was performed to examine the effect of *DCTPP1*-knockdown on cell proliferation *in vitro* by using CCK-8 detection kit. (**c**) Soft agar colony assays were performed and representative images from the assays were shown (upper panel). Statistical plots were conducted from >3 randomly selected fields (lower panel). (**d**) Cell cycles of MCF-7 cells were determined by flow cytometry and statistical results were indicated to compare the effects of DCTPP1 expression on cell cycle. (**e**) The overexpression of *DCTPP1* in transfected MDA-MB-231 cells was confirmed by immunoblotting (left panel) and real-time PCR (right panel). (**f**) CCK-8 assay was performed to detect the proliferation rate of DCTPP1 overexpressing and control cells. All the values shown were represented as the means±s.d. (***P⩽0.001, **P⩽0.01).

To further elucidate the significance of DCTPP1 in tumorigenesis, after screening of DCTPP1 expression in a panel of cell lines (Supplementary Figure 1) we established two *DCTPP1* stable knockdown MCF-7 cell lines by transfecting vectors containing short hairpin RNA (shRNA) specific to *DCTPP1*. After puromycin selection and clonal expansion, a significant reduction of DCTPP1 expression in two *DCTPP1*-shRNA-transfected MCF-7 cell lines (MCF-7-shRNA1 and MCF-7-shRNA2) was detectable with >60% of the inhibitory efficacy (Figure 2a).

We further investigated the effects of *DCTPP1* knockdown on cell proliferation. Compared with control shRNA-transfected MCF-7 cells (MCF-7-NC), the *in vitro* growth rates of MCF-7-shRNA1 and MCF-7-shRNA2 cells were both decreased significantly (Figure 2b). In addition, results from soft agar colony formation assay indicated that colony formation ability also decreased markedly by 59.7% and 53.2%, respectively, when DCTPP1 was downregulated by shRNA1 and shRNA2 in MCF-7 cells (Figure 2c). In cell cycle analysis, the percentage of S phase markedly decreased in *DCTPP1*-shRNA-transfected MCF-7 cells by 24.1% and 20.8%, respectively, when compared with control shRNA-transfected cells (*P*<0.05), whereas the cell population in G1 phase increased from 48.65 to 57.4% and 56.6% in MCF-7-shRNA1 and MCF-7-shRNA2 cells, respectively (Figure 2d). These results support that the knockdown of *DCTPP1* reduces the growth of MCF-7 cells and alters cell cycle *in vitro*.

We also constructed *DCTPP1* stably overexpressed breast cancer cell line MDA-MB-231, which expressed low level of DCTPP1 originally (Supplementary Figure 1). DCTPP1 was markedly higher expressed after stable transfection (Figure 2e). However, no significant difference in *in vitro* proliferation was observed after DCTPP1 overexpression (Figure 2f). Meanwhile, colony formation capacity and cell cycle exhibited little change when DCTPP1 was highly expressed (data not shown).

### Silencing *DCTPP1* attenuates mammosphere formation capacity of breast cancer cell lines

**Figure 3:**
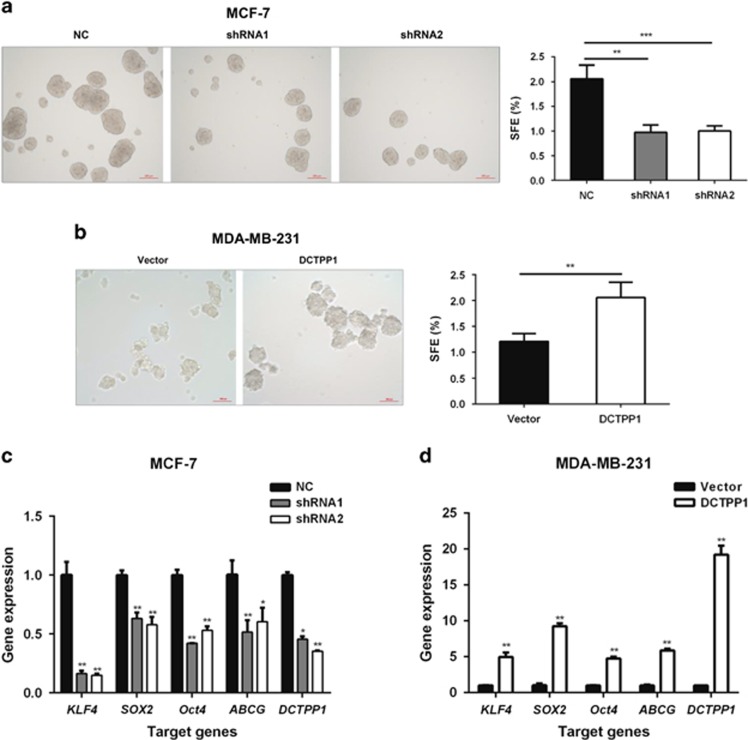
DCTPP1 promotes mammosphere formation in breast cancer cells. (**a**) Mammospheres from NC-shRNA-transfected MCF-7 and two *DCTPP1*-shRNA-transfected MCF-7 cells were observed under light microscope with × 100 magnification (Scale bar, 200 μm; left panel). The sphere formation percentage was calculated from randomly selected eight fields and analyzed by Graphpad Prism 6 (right panel). (**b**) Mammospheres from *DCTPP1* overexpressing and control MDA-MB-231 cells were observed at day 5 after conditional induction. The spheres markedly enlarged with *DCTPP1* overexpression (left panel). The sphere formation percentages were calculated from randomly selected eight fields and analyzed by Graphpad Prism 6 (right panel). The transcription factors related to CSC properties were measured by real-time PCR as well as DCTPP1 in *DCTPP1*-deficient MCF-7 (**c**) and *DCTPP1* overexpressing MDA-MB-231 cells (**d**). (***P⩽0.001, ** *P*⩽0.01)

It has been reported that breast cancer stem cells (CSCs) could be enriched in suspension cultures as mammospheres,^31^ which is used for functional characterization of stem-cell like population with vigorously tumor-initiating potential.^32^ To determine the possible involvement of DCTPP1 in maintaining CSC-like property, mammosphere formation capacity was determined in *DCTPP1* knockdown and overexpressed cell lines in parallel accordingly.^33^ Notably, *DCTPP1* silencing in MCF-7 resulted in a significant reduction in both sphere size and sphere forming number (Figure 3a). In *DCTPP1*-overexpressed MDA-MB-231 cells, on the contrary, the sphere size and forming number showed apparent increase when compared with the control cells (Figure 3b). We measured the key transcription factors involved in CSC maintenance such as *KLF4*, *SOX2*, *Oct4*^34^ and drug resistant gene *ABCG2*^35^ in the mammospheres. Results from real-time PCR indicated that with the altered mammosphere formation capacity and the percentage of CSC-like cells in mammospheres, all four genes decreased in *DCTPP1*-knockdown MCF-7 (Figure 3c) and increased in *DCTPP1*-overexpressed MDA-MB-231 mammosphere cells (Figure 3d). For instance, *KLF4* expression was decreased to 17% after *DCTPP1* knockdown, but increased more than five fold with *DCTPP1* overexpression. These results thus demonstrate that DCTPP1 is also engaged in promoting mammosphere formation.

### Sequence similarity with other members of all-α NTP-PPase superfamily and homology modeling of DCTPP1

**Figure 4:**
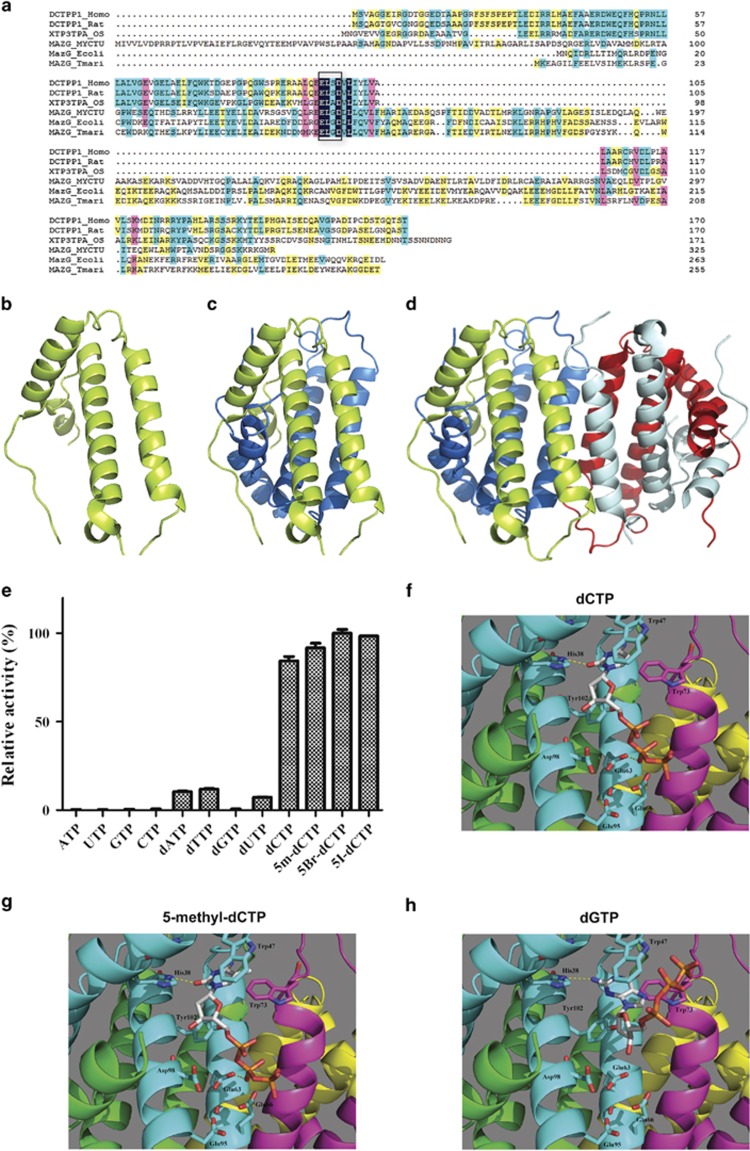
DCTPP1 possesses MazG-like tetrameric domain through structure simulation and specifically catalyzes dCTP and its derivatives with structure complementarity. (**a**) *DCTPP1* sequences from human, Rat, Oryza sativa and MazG proteins from *E. coli*, *M.tb* and *Thermotoga maritima* were aligned by DNAman software. The amino-acid residues with 100%, ⩾75%, ⩾50%, ⩾33% homolog were shown with indigo, pink, azure and yellow background, respectively. The EXXD motif was indicated by black frame. The 3-dimensional models of human DCTPP1 in monomeric (**b**), dimeric (**c**) and tetrameric (**d**) were generated by PyMol software. Protein monomer in tetrameric form was shown in limon, marine blue, pale cyan and red cartoons. The enzymatic activity of purified recombinant DCTPP1 was measured by using 200 μM (d)NTP substrates indicated (**e**). Binding modes of 5-methyl-dCTP (**f**), dCTP (**g**) and dGTP (**h**) to the human DCTPP1 tetramer were predicted by GLIDE software. Tetrameric DCTPP1 protein was composed by four monomers indicated in cyan, magenta, green and yellow cartoons, respectively. Oxygen atoms of dNTPs were shown in red and nitrogen atoms in blue. Carbon and phosphorus atoms of dNTPs were shown in white and orange, respectively. Crucial residues in the binding site were shown as stick and labeled. Hydrogen bonds are depicted in dotted line in yellow. Figures were generated by PyMOL Molecular Graphics System (DeLano Scientific, San Carlos, CA, USA).

All the above results indicate that DCTPP1 is involved in the promotion of tumorigenesis as well as the maintenance of CSC properties. As a member of NTP-PPases, how this biochemical enzyme participates in the pathological processes is worthy of further exploration. Therefore, we first examined the biochemical characters of DCTPP1. Sequence similarity was aligned between members of all-α NTP-PPase superfamily. The results showed that DCTPP1 from eukaryocytes shared similar homology with MazG protein from prokaryocyte organisms (Figure 4a). Furthermore, DCTPP1 retained key amino-acid motif ExxD required for Mg^2+^ binding and substrate coordination, which was consistent with other members of MazG-like family (Figure 4a). Secondary structure prediction and homology modeling were also performed based on the crystal structure of mouse DCTPP1 proteins RS21-C6 which shared 75.9% sequence similarity to human DCTPP1.^36^ The modeling results revealed that human DCTPP1 contained four α-helices (Figure 4b). Four monomeric molecules of DCTPP1 formed an asymmetric dimer of dimers (Figures 4c and d), which was highly similar to the crystal structure of mouse DCTPP1^36^ and other members of all-α NTP-PPase family.

Although previous study reported that one typical MazG-like core domain consisted of five α-helices,^37^ our results suggest that DCTPP1 retain a minimal set of helices sufficient for the formation of the active enzymatic sites during evolution.

### Human DCTPP1 exhibits substrate preference to dCTP and structurally similar derivates including 5-methyl-dCTP and 5-halo-dCTP

To reveal the biochemical properties, human DCTPP1 was prokaryotically expressed and purified by Ni-NTA affinity chromatography with expected 21 kDa molecular weight containing a C-terminal 6 × His and myc tag (Supplementary Figure 2B). The enzymatic activity of DCTPP1 was measured by PPi quantification hydrolyzed from (d)NTP substrates. PPi was converted to Pi by inorganic pyrophosphatase and the Pi level was subsequently quantified by an enzyme-coupled colorimetric method. As DCTPP1 possessed the conserved Mg^2+^ binding EXXD motif (Figure 4a), DCTPP1 performed the optimized enzymatic activity with Mg^2+^ as the cofactor among the four divalent cations tested (Supplementary Figure 2B).The catalytic activity was dependent on the concentration of Mg^2+^ (Supplementary Figure 2C). The effects of pH on DCTPP1 stability were also examined. DCTPP1 exhibited >80% of its activity between pH 7.8 and 11.0 with the optimal pH value at 8.7 (Supplementary Figure 2D).

The substrate specificity of purified DCTPP1 was determined through using canonical and noncanonical (d)NTPs as substrates. The enzymatic parameters for different substrates were shown in Table 2. Whereas MazG from *Mtb* hydrolyzes all the eight canonical (d)NTPs with preference for GTP and dGTP, DCTPP1 was more specific for dCTP with the *K_m_* value of 152.3±10.91 μM when compared with other four dNTPs. However, DCTPP1 exhibited very low substrate affinity with the four canonical NTPs (Figure 4e).

Since the mouse RS21-C6, a highly conserved ortholog of the human DCTPP1, is capable of hydrolyzing 5-methyl-dCTP with *K_m_* value of 48.5 μM,^38^ we subsequently tested the hydrolysis activity of DCTPP1 toward structurally similar substrates, including 5-methyl-dCTP and halogen-modified dCTP. Notably, DCTPP1 not only hydrolyzed 5-methyl-dCTP with relatively higher specificity than dCTP (*K_m_*= 85.03±7.02 μM), but also exhibited considerably high hydrolyzing activity for 5-halogenated dCTPs (*K_m_* value of 108.9±6.48 μM for 5-Br-dCTP and 94.40±5.20 μM for 5-I-dCTP; Table 2). This was consistent with the results from mouse RS21-C6 that it showed highest pyrophosphatase activity against 5-I-dCTP and 5-Br-dCTP in the previous research.^38^

To verify the enzymatic results from the structural viewpoint, we also conducted the computational docking by using GLIDE (Grid-based ligand docking from energetics software).^39^ The results indicated that the cytosines of 5-methyl-dCTP and dCTP bound deeply into the substrate pocket, which was composed of the residues from the dimmer interface, including His^38^, Trp^47^, Glu^63^, Glu^66^, Trp^73^, Glu^95^, Asp^98^ and Tyr^102^. There were two important hydrogen bonds for binding dCTP and 5-methyl-dCTP, which were formed between the oxygen atoms of cytosines and His^38^, and Glu^63^ OE2 and the O-1B atom of triphosphates. The side chain of Tyr^102^ formed hydrophobic interactions with the deoxyriboses. Furthermore, Trp^47^ and Trp^73^ formed an extra hydrophobic contact with 5-methyl-cytosine comparing to cytosine. This might be why DCTPP1 catalyzed 5-methyl-dCTP more efficiently than dCTP based on our own enzymatic data (Figures 4f and g, Table 2). Although there was also a hydrogen bond between the guanosine of dGTP and His^38^, the triphosphate was flipped out of active site. The catalytic residues could not form contacts with it (Figure 4h). As a consequence, DCTPP1 could not hydrolyze the dGTP. These results indicate that DCTPP1 possesses (deoxy)ribonucleoside triphosphatase activity that exhibits substrate preference to dCTP and its derivates.

### DCTPP1 modulates the intracellular concentration of 5-methyl-dCTP

**Figure 5:**
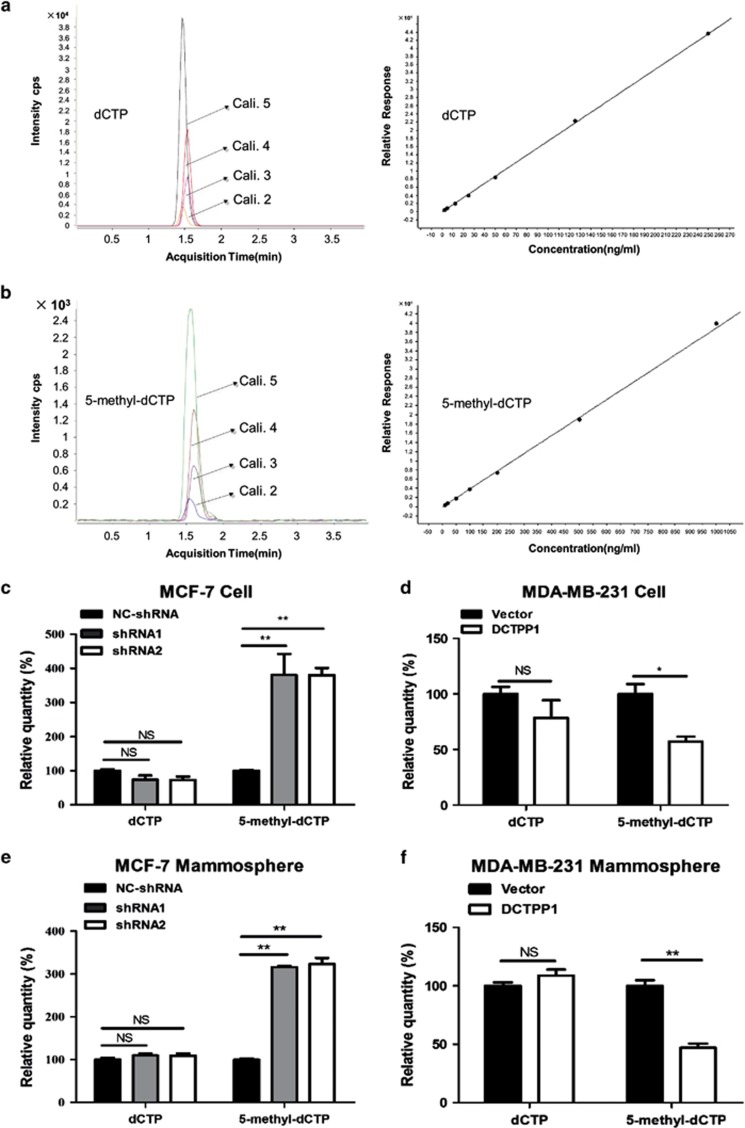
DCTPP1 modulates intracellular 5-methyl-dCTP concentration. The concentration of intracellular dCTP and 5-methyl-dCTP was determined by LC-MS assay based on the concentration of internal standards. Representative MRM chromatograms of the dCTP calibrators (**a**, left panel) and 5-methyl-dCTP calibrators (**b**, left panel) was shown.The calibration curves of dCTP and 5-methyl-dCTP were shown in right panel of (**a** and **b**), respectively. The concentration of dCTP and 5-methyl-dCTP in *DCTPP1*-deficient MCF-7 cells (**c**) and overexpressed MDA-MB-231 cells (**d**) was calculated accordingly. The concentration of dCTP and 5-methyl-dCTP in mammospheres formed by *DCTPP1* knockdown MCF-7 (**e**) and *DCTPP1*-overexpressed MDA-MB-231 cells (**f**) were also measured by LC-MS assays. All data were collected from at least three independent experiments and each experiment was performed in triplicate.

Considering DCTPP1 hydrolyzing dCTP and 5-methyl-dCTP in cell-free enzymatic assay, the effect of DCTPP1 on intracellular dCTPs metabolism was further analyzed by liquid chromatography coupled with tandem mass spectrometry (LC-MS) using internal standard (Figures 5a and b, left). Standard curves of dCTP and 5-methyl-dCTP were established according to the area of peaks with different concentrations of standard nucleotide triphosphates. The quantity of dCTP and 5-methyl-dCTP from the unknown samples were calculated accordingly (Figures 5a and b, right). When we compared the cellular concentrations of these two dNTPs, the quantity of 5-methyl-dCTP increased by about 2.8 times in two *DCTPP1*-knockdown MCF-7 cells than in the control cells (Figure 5c). Consistently, the intracellular 5-methyl-dCTP decreased by 42.7% in MDA-MB-231 cells with *DCTPP1* overexpression. Even in mammosphere cells, the intracellular 5-methyl-dCTP increased with *DCTPP1* knockdown, whereas decreased in *DCTPP1*-overexpressed mammospheres with statistical significance. These results indicate that DCTPP1 can affect the intracellular quantity of 5-methyl-dCTP with marked efficiency in cellular level, which probably results from the catalytic priority of DCTPP1 to 5-methyl-dCTP. However, there were no obvious variations of dCTP concentration in *DCTPP1* knockdown or overexpressed cells (Figure 5d), which is probably due to other regulation mechanisms on dCTP in cells.

### DCTPP1 alters global DNA methylation level in breast cancer cells

**Figure 6:**
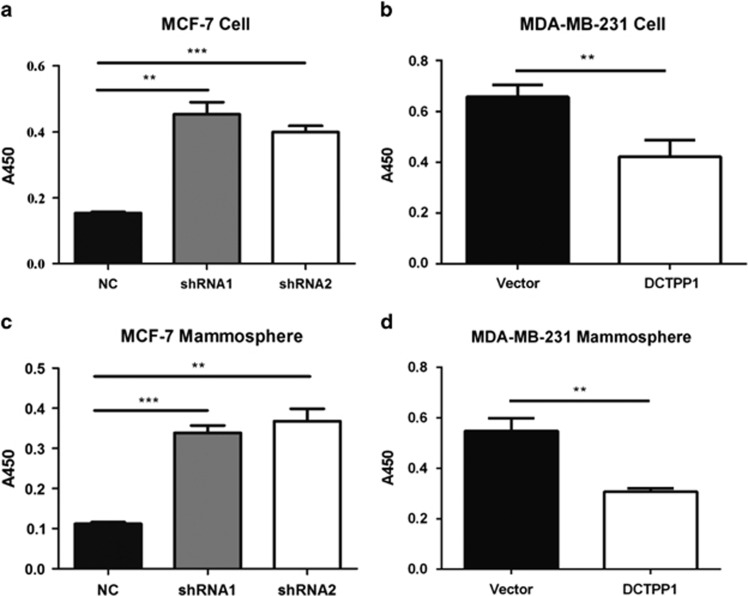
DCTPP1 alters the global DNA methylation level in breast cancer cells. (**a**) Comparison of global DNA methylation levels between MCF-7 cells transfected with control and *DCTPP1*-specific shRNAs containing vectors. The data were obtained from three independent experiments. (**b**) The global DNA methylation in MDA-MB-231 cells with or without *DCTPP1* overexpression was also compared. Global DNA methylation levels in mammospheres formed by MCF-7 cells (**c**) and MDA-MB-231 cells (**d**) were quantitatively compared. All the data were presented as means±s.d. from three independent experiments. (*P⩽0.05, **P⩽0.01, ***P⩽0.001)

Given that the extraordinary concentration of intracellular 5-methyl-dCTP might increase its incorporation into DNA leading to aberrant gene methylation and gene expression,^40, 41, 42^ we speculated that DCTPP1 might alter the DNA methylation level. To address this possibility, we determined the global DNA methylation in *DCTPP1* knockdown MCF-7 cells and overexpressed MDA-MB-231 cells. As shown in Figure 6a, the global methylation level was markedly elevated in two *DCTPP1* knockdown MCF-7 cell lines with equal input of genomic DNA. Consistent with the increased methylation in *DCTPP1*-deficient MCF-7 cells, mammospheres formed by *DCTPP1*-deficient MCF-7 cells also displayed elevated global DNA methylation (Figure 6c). MDA-MB-231 cells and its mammospheres, however, showed reduced global DNA methylation with the transfection of DCTPP1 (Figures 6b and d). With the similar expression level of DNMT1 (DNA methyltransferase 1) regardless of DCTPP1 expression (Supplementary Figures 3A and B), DCTPP1 was thus demonstrated to participate in the regulation of global DNA methylation, partially through the modulation of dCTP metabolisms.

## Discussion

The first NTP-PPase, MutT, was identified by Bhatnagar and Bessman in 1988 from *E. Coli* with low substrate specificity to dGTP and other noncanonical NTPs. Until now NTP-PPases are demonstrated to prevalently distribute from prokaryote organisms (such as *E.coli*, fungi and virus) to mammalians.^6^ In the present study, we characterized a novel NTP-PPase in human known as DCTPP1 whose biological function was poorly investigated before. We have verified the elevated expression of DCTPP1 in breast cancer (Figure 1). More significantly, DCTPP1 expression was obviously related to higher tumor stage and grade (Table 1) and exhibited strong correlation with lower overall survival probability and a poor prognosis in breast cancer patients (Figure 1d).This not only provided clinical evidence for DCTPP1 to be engaged in cancer progression, but also might make DCTPP1 become an additional prognosis biomarker in breast cancer.

DCTPP1 expression affects cell proliferation as well as cell cycle *in vitro*. In our study, DCTPP1 deletion increased the percentage of G1 phase, whereas it decreased the percentage of S phase. During mitosis, one critical cell-cycle control is G1 checkpoint where cells with out-of-order DNA are prevented from entering the S phase.^43^ Targeting the noncanonical dCTPs, DCTPP1 modulates the concentration of noncanonical dCTPs in nucleotide pools. Low expression of DCTPP1 in MCF-7 cells might reinforce the incorporation of noncanonical nucleotide with higher incidence and lead to the increased mutations in DNA and the subsequent G1-S arrest. At present, it is still unclear whether and how cells sense the abnormal nucleotides in nucleotide pools and affect the DNA synthesis and late-on mitosis. It is reasonable that the brake of these processes under *DCTPP1*-deficient condition might be a self-protection mechanism to guarantee the passage of normal daughter cells with competitive priority. Especially under tumorigenesis, it is necessary to maintain the correct DNA replication to fulfill the massive requirement for the high rate of proliferation. In addition, the capacity of mammosphere formation also unexpectedly reduced in *DCTPP1-*downregulated MCF-7 cells and increased in *DCTPP1*-overexpressed MDA-MB-231 cells. How the alteration of intracellular nucleotide composition affects the maintenance of CSC-like property needs to be further investigated.

Through enzyme kinetics analysis, DCTPP1 displayed high substrate specificity to dCTP and its structural homologs such as 5-methyl-dCTP and 5-halo-dCTPs, whereas little activity against NTPs (Table 2), which is similar to the results from Requena's group.^29^ This was further validated both through the computer modeling that 5-methyl-dCTP matched best with the active binding site of DCTPP1 and, more importantly, by the results from the determination of intracellular 5-methyl-dCTP concentration by LC-MS assay. The highest affinity of DCTPP1 to 5-methyl-dCTP arouses great interest in that as the fifth nucleotide 5-methyl-dCTP has long been associated with DNA methylation and transcriptional silencing.^44^ Although DNA methylation is thought to be an enzyme-mediated post-replication process,^45^ there are evidences that endogenous 5-methyl-dCTP can be incorporated into DNA, leading to gene silencing^40, 46^ and developmental arrest.^47^ Not only 5-methyl-dCTP, 5-chlorocytosine was also demonstrated to be incorporated into DNA to alter the DNA methylation patterns in tumors.^48^ In addition, it was reported recently that 5-methyl-deoxycytidine could enhance the substrate activity of DNA polymerase *in vitro* with the more incorporation of 5-methyl-dCTP in DNA.^49^ In other words, abnormal DNA methylation might also result from extra incorporation of 5-methyl-dCTP into DNA strand during replication. However, it remains unclear whether there is a preventive mechanism against this replication abnormalty. Here we advanced the understanding of DCTPP1 function by linking this enzyme to aberrant intracellular 5-methyl-dCTP metabolism and global DNA methylation. With the increase of 5-methyl-dCTP in *DCTPP1*-deficiency MCF-7 cells, the global DNA methylation increased nearly threefolds when compared with control cells. *DCTPP1* overexpression in MDA-MB-231 cells led to the decrease of both intracellular 5-methyl-dCTP and global methylation significantly. In fact, with the elevated expression of DCTPP1 in cancerous regions, we also observed the hypomethylation in tumor tissues of gastric cancer (data not shown). It is worth noting that the global DNA hypomethylation was first revealed in 1983^50^ and is the most common molecular lesion in cancer cells^51^ as well as tumor-initiating cells.^52^ In addition, in the previous^28^ and present studies we found that DCTPP1 was inclined to accumulate in the nucleus of cancer cells compared with the paired adjacent tissue cells in multiple carcinomas, which facilitated the regulation of nucleotide concentration. The observations are in line with the 'house-cleaning' function of NTP-PPases, suggesting that DCTPP1 could conceivably act to degrade the intracellular methylated dCTP and reduce the aberrant DNA methylation subsequently. Although it is still far from the conclusion that this is a universal mechanism, we still proposed a novel mechanism of aberrant hypomethylation through the modulation of dCTP/ 5-methyl-dCTP metabolism by DCTPP1 based on our study.

More interestingly, DCTPP1 can also hydrolyze 5-halo-dCTPs (including 5-Br-dCTP and 5-I-dCTP) with high efficacy in our study. Under inflammation, a panel of oxidized and halogenated damage products in living cells is generated to cause damages to host DNA.^53, 54^ Among them are particularly sinister damage products, 5-Cl-dCTP^55, 56^ and 5-Br-dCTP.^48^ They belong to cytosine damage products generated by activated neutrophils and eosinophils in chronic inflammatory environment.^48,53^ They can mimic 5-methyl-dCTP and induce inappropriate methylation within the CpG sequence by altering the selectivity of human DNMT1.^57^ Incorporation of 5-Cl-dCTP into mammalian cells results in heritable gene silencing and the alteration of methylation pattern as well.^58^ In the *in vitro* studies, it has been demonstrated that 5-halo-dCTP was recognized as 5-methyl-dCTP by methylation-sensitive DNA-binding proteins.^59^ Therefore, cytosine damage analog is supposed to provide a bridge between inflammation and aberrant methylation, which is related to abnormal gene expression and cancer development. However, there is no specific repairing mechanism identified for 5-halo-dCTP to date.^60^ Bearing the catalytic activity to 5-halo-dCTPs, DCTPP1 could be considered as a pyrimidine-specific 'house-cleaning' enzyme to maintain nucleotide homeostasis under inflammation. Thus it will be similar to mycobacterial homology MazG that degrades 5-OH-dCTP *in vivo* and safeguards the genetic stability in bacteria.^15^

Our report here provides the first quantitative evidence to prove the house-cleaning roles of *DCTPP1* at cellular level. On the basis of our findings, we conclude that DCTPP1 acts as an intracellular modulator for 5-methyl-dCTP metabolism and global hypomethylation, which is engaged in promoting cancer cell proliferation and stemness properties. Its potential role in antitumor activities can be achieved by using a potent natural product triptolide that directly inhibits DCTPP1 pyrophosphatase activity.^61^ DCTPP1 can thus be potentially used as a novel target for cancer diagnosis and therapy in the future.

## Materials and methods

### Cell lines

The breast cancer cell lines MCF-7 and MDA-MB-231 and other cell lines used in the DCTPP1 expression screening were purchased from the Shanghai Institute for Biological Sciences, Chinese Academy of Sciences, routinely maintained in the laboratory and cultured in Dulbecco's modified Eagle's medium (HyClone, Logan, UT, USA) supplemented with 10% fetal bovine serum (Invitrogen, Carlsbad, CA, USA).

### Breast cancer tissue microarray

The study protocol was approved by the Ethics Committee of Shanghai Jiao Tong University School of Medicine. Tissue microarray containing 161 formalin-fixed, paraffin-embedded breast cancer tissues and 132 paired adjacent tissues was purchased from the Shanghai Outdo Biotech Company (Shanghai, China). All the tissue samples were collected with signed informed consent according to the internal review. Each core of representative areas from tumor and normal breast tissue (2.0 mm in diameter and 4 μm in thickness) were prepared according to a standard method. Clinicopathological stage and grade were assigned according to the criteria from the Union for International Cancer Control and the World Health Organization. Immunohistochemistry was performed according to the methods described in the 'Supplementary material'. DCTPP1 expression was evaluated independently by two pathologists in a blinded manner using the Allred scoring system.^62^ For each section, five fields were randomly selected. The staining was scored according to the percentage of DCTPP1-positive cells. For data analysis, scores >50 were considered as high expression.

### Kaplan–Meier plotter web tool

Expression array data were evaluated using the Kaplan–Meier Plotter version 2014 (http://www.kmplot.com) as described.^63^ Data sets included gene expression and survival data from the Gene Expression Omnibus (Affymetrix HG-U133A and HG-U133 Plus 2.0 microarrays). The DCTPP1 (*218069_at*) probe set was used. Query parameters included overall survival and recurrence-free survival, split patients by median, auto-select best cutoff and follow-up threshold of all. DCTPP1 probe expression range was 159–5440, cutoff value was 1954 and the hazard ratio and log-rank *P* significance values were calculated via the website interface.

### Homolog structure modeling of human DCTPP1

The 3D models of human DCTPP1 and its 5-methyl-dCTP-bound tetramer were generated using MODELLER software (version 9.12; Sali and Blundell 1993)^64^ based on the X-ray crystal structure of the tetrameric mouse dCTPase 1 (Protein Data Bank entry 2OIG, resolution 3.3Å).^36^ In the docking process, GLIDE software^39^ was used for predicting dCTP and dGTP binding poses based on the 5-methyle-dCTP-bound tetramer structure model. Detailed procedures were summarized in 'Supplementary material'.

### Catalytic activity assay of DCTPP1

Cloning, expression and purification of DCTPP1 recombinant protein was described in the 'Supplementary material' for catalytic activity assay. The substrates of DCTPP1 tested in this study contained canonical and noncanonical (d)NTPs. Detailed procedures were summarized in 'Supplementary material'.

Measurement of intracellular dCTP and 5-methyl-dCTP by LC-MS analysis; cells were harvested with 0.25% (w/v) trypsin solution containing 0.04% (w/v) EDTA and counted twice with Thomas counting chamber (Thomas Co., Philadelphia, PA, USA). Cells were washed twice with cold phosphate-buffered saline and resuspended in precold 60% methanol, vortexed vigorously for 15 s and immediately frozen in liquid nitrogen. Sample solutions were stored at −80 °C for lysis of cell membranes. After 24 h, sample solutions were thawed on the ice, vortexed vigorously and centrifuged at 15,000 r.p.m. for 10 min at 4 °C. The supernatants were collected and stored at −80 °C for LC-MS analysis. LC-MS assay was performed using an Agilent 1200 HPLC system coupled to an Agilent 6410 triple quadruple mass spectrometer (Agilent Technologies, Palo Alto, CA, USA) where dCTP^13^C,^15^N and dGTP^13^C,^15^N were used as internal standards for quantification of dCTP and 5-methyl-dCTP, respectively. The data analysis was processed using the MassHunter software package (Agilent Technologies). Detailed procedures of LC-MS assay were summarized in 'Supplementary material'.

### Global DNA methylation assay

The genomic DNA was extracted from cells by using universal genomic DNA extraction kit (Takara, Dalian, China) according to the manufacturer's instruction. The DNA concentration was measured by NanoDrop 2000 (Thermo Scientific, Hudson, NH, USA). The global DNA methylation was assayed by Imprint Methylated DNA quantification kit (Sigma-Aldrich, St Louis, MO, USA) according to the protocols provided.

### Statistical analysis

Statistical analyses were performed with SPSS version 20.0 (SPSS Inc, Chicago, IL, USA). The relationships between DCTPP1 expression and clinicopathological parameters were analyzed using a two-tailed *χ^2^*-test. Survival curves were plotted using the Kaplan–Meier method and compared using the log-rank test. For most of the *in vitro* experiments, Student's *t*-tests were used to calculate the *P*-value. Data in Figures 2, 3, 5, 6 were collected from at least three independent experiments and each experiment was performed in triplicate. All the values shown were represented as the means±s.d. *P*<0.05 was considered significantly different.

## Figures and Tables

**Table 1:** Correlation between clinicopathological features and DCTPP1 expression in breast cancer tissue

	*DCTPP1 expression*				*Total*	*χ*^*2*^	P*-value*
*Variables*	⩽*25 * N *(%)*	⩽*50 *N *(%)*	⩽*75 *N *(%)*	⩽*100 *N *(%)*	*161*		
*Age*						5.744	0.125
⩽60	38 (33.0)	42 (36.5)	20 (17.4)	15 (13.0)	115 (100.0)		
60	8 (18.2)	15 (34.1)	10 (22.7)	11 (25.0)	44 (100.0)		
							
T *cassification*						27.151	**0.001**
*T*1	22 (50.0)	16 (36.4)	5 (11.4)	1 (2.3)	44 (100.0)		
*T*2	14 (17.9)	29 (37.2)	20 (25.6)	15 (19.2)	78 (100.0)		
*T*3	7 (28.0)	7 (28.0)	3 (12.0)	8 (32.0)	25 (100.0)		
*T*4	1 (25.0)	3 (75.0)	0 (0.0)	0 (0)	4 (100)		
							
N *Classification*						7.519	0.583
*N*0	19 (24.7)	29 (37.7)	18 (23.4)	11 (14.3)	77 (100)		
*N*1	12 (36.4)	11 (33.3)	4 (12.1)	6 (18.2)	33 (100)		
*N*2	11 (34.3)	14 (43.8)	3 (9.4)	4 (12.5)	32 (100)		
*N*3	4 (25.0)	4 (25.0)	4 (25.0)	4 (25.0)	16 (100)		
							
*Tumor grade*						15.261	**0.018**
I	0 (0)	1 (50.0)	1 (50.0)	0 (0)	2 (100.0)		
II	30 (38.5)	26 (33.3)	12 (15.4)	10 (12.8)	78 (100.0)		
III	4 (9.5)	15 (35.7)	14 (33.3)	9 (21.4)	42 (100.0)		
							
*Histological type*						15.995	0.382
Intraductal	1 (50.0)	1 (50.0)	0 (0)	0 (0)	2 (100.0)		
Invasive ductal	36 (27.3)	48 (36.4)	27 (20.5)	21 (15.9)	132 (100.0)		
Lobular	5 (41.7)	3 (25.0)	1 (8.3)	3 (25.0)	12 (100.0)		
Medullary	0 (0)	1 (100)	0 (0)	0 (0)	1 (100.0)		
Mucinous	4 (40.0)	5 (50.0)	1 (10.0)	0 (0)	10 (100.0)		
Indeterminate	0 (0)	0 (0)	2 (50)	2 (50)	4 (100)		
							
*Clinical stage*						9.094	0.168
I	12 (40.0)	13 (43.3)	4 (13.3)	1 (3.3)	30 (100.0)		
II	15 (23.8)	20 (31.7)	16 (25.4)	12 (19.0)	63 (100.0)		
III	17 (29.3)	22 (37.9)	8 (13.8)	11 (19.0)	58 (100.0)		
							
*ER presence*						0.747	0.862
Positive	15 (23.4)	26 (40.6)	14 (21.9)	9 (14.1)	64 (100)		
Negative	10 (29.4)	11 (32.4)	8 (23.5)	5 (14.7)	34 (100)		
							
*PR presence*						4.824	0.185
Positive	15 (25.4)	27 (45.8)	11 (18.6)	6 (10.2)	59 (100)		
Negative	10 (26.3)	10 (26.3)	10 (26.3)	8 (21.1)	38 (100)		
							
*HER-2 presence*						4.23	0.234
Positive	14 (31.1)	19 (42.2)	7 (15.6)	5 (11.1)	45 (100)		
Negative	10 (19.2)	18 (34.6)	15 (28.8)	9 (71.3)	52 (100)		

Abbreviations: DCTPP1, dCTP pyrophosphatase 1; N, number.

**Table 2:** Kinetic parameters of DCTPP1 protein

*Substrate*	*V*_*max*_	*k*_*cat*_	*K*_*m*_	*K*_*cat*_*/K*_*m*_
*(d)NTP*	*nmol/min/μg*	*min*^*-1*^	*μM*	*min*^*-1*^*mM*^*-1*^
ATP	NR	NR	NR	NR
UTP	NR	NR	NR	NR
GTP	NR	NR	NR	NR
CTP	NR	NR	NR	NR
dATP	0.39±0.01	8.49±0.27	292.8±18.07	28.99
dTTP	0.30±0.01	6.49±0.22	196.10±16.27	33.09
dGTP	NR	NR	NR	NR
dUTP	0.22±0.01	4.86±0.15	240.6±19.07	20.20
dCTP	1.63±0.05	22.02±0.43	152.3±10.91	144.58
5-Methyl-dCTP	0.99±0.03	21.70±0.60	85.03±7.02	255.20
5-Br-dCTP	1.38±0.03	30.28±0.66	108.9±6.48	278.05
5-I-dCTP	1.18±0.02	25.83±0.05	94.40±5.20	273.62

Abbreviation: NR, no reaction.

Standard assay was conducted as described in 'Materials and methods'. The results were analyzed from the reactions that contained nine different concentrations of each substrate. All data were presented as mean±s.d. from at least three independent experiments and each experiment was performed in triplicate. The bold values represent DCTPP1 expression is associated with corresponding clinical parameters.

## Notes

The authors declared no conflict of interest.
