# Supplementary figures and images for: Human dCTP pyrophosphatase 1 promotes breast cancer cell growth and stemness through the modulation on 5-methyl-dCTP metabolism and global hypomethylation
Source: Oncogenesis. 2015 Jun 15;4(6):e159–. doi: 10.1038/oncsis.2015.10 (PMC4491611; doi:10.1038/oncsis.2015.10)

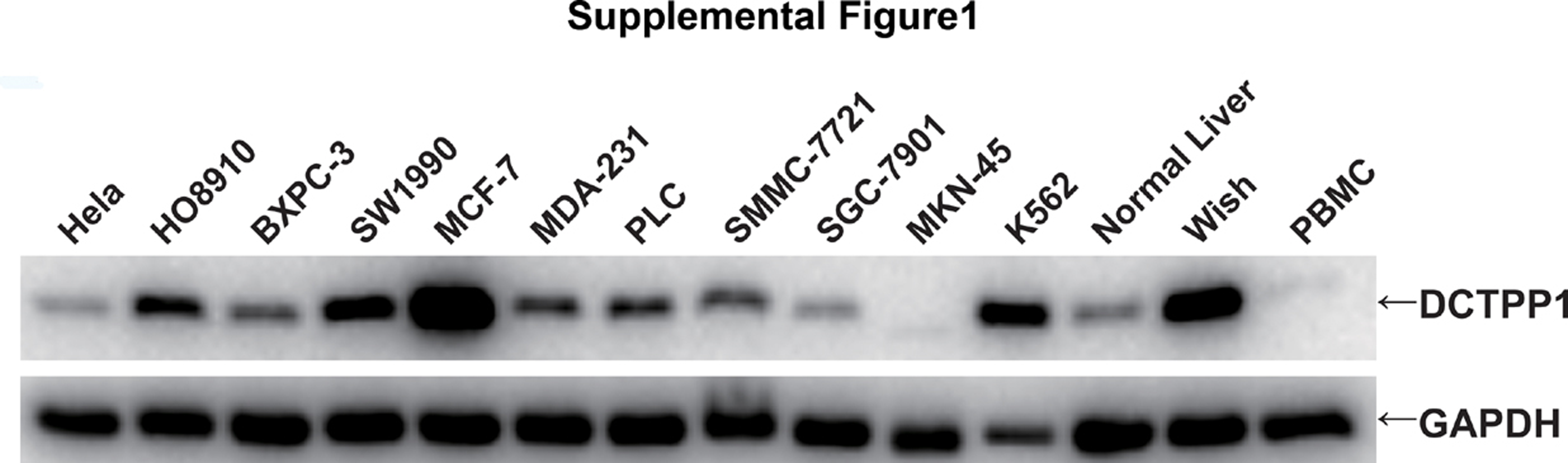

Supplement: Supplementary Figure 1 [file oncsis201510x1.tif]

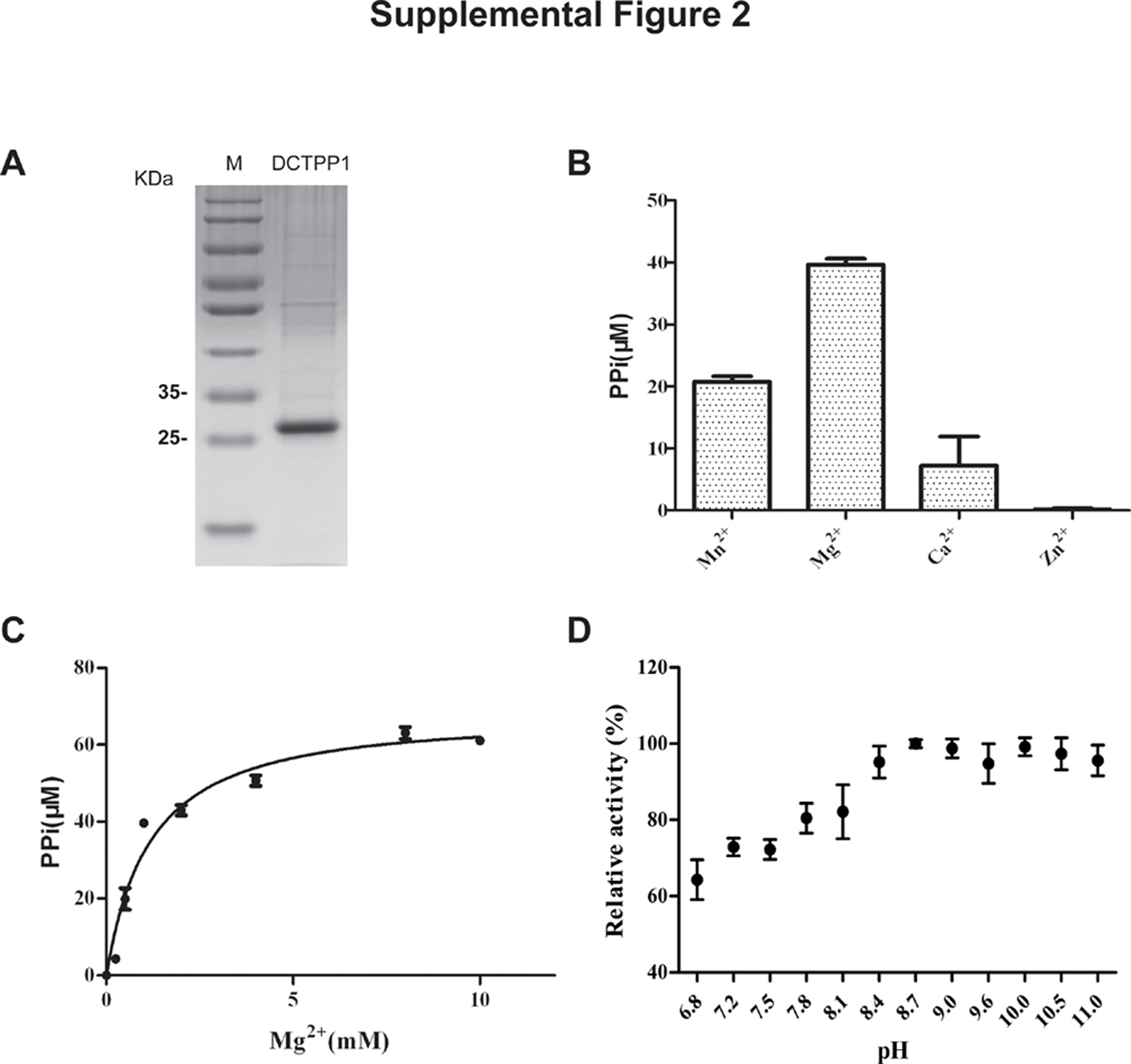

Supplement: Supplementary Figure 2 [file oncsis201510x2.tif]

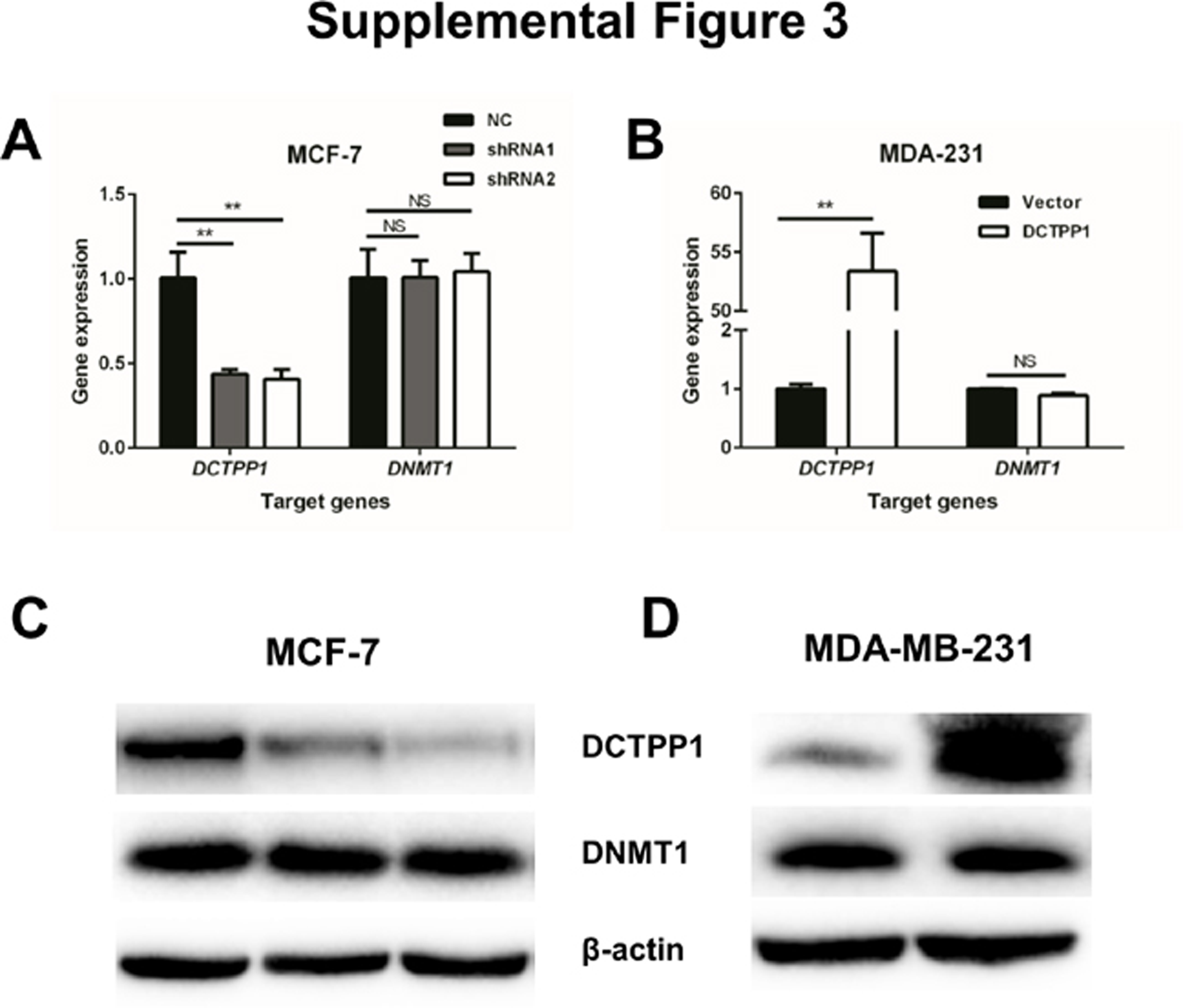

Supplement: Supplementary Figure 3 [file oncsis201510x3.tif]
