# Supplementary material for: Human dCTP pyrophosphatase 1 promotes breast cancer cell growth and stemness through the modulation on 5-methyl-dCTP metabolism and global hypomethylation
Source: Oncogenesis. 2015 Jun 15;4(6):e159–. doi: 10.1038/oncsis.2015.10 (PMC4491611; doi:10.1038/oncsis.2015.10)
Supplement: Supplementary Information [file oncsis201510x4.doc]

**Supplemental materials**

**Materials and Methods**

**Main reagents**

Restriction enzymes and Pfu DNA polymerase were purchased from Takara (Dalian, China). T4 DNA ligase and the canonical (d)NTPs were obtained from Fermentas (St. Leon-Rot, Germany). The non-canonical (d)NTPs used as substrates for DCTPP1 and standards molecules in LC-MS measurement were purchased from Sigma-Aldrich (St Louis, USA), Jena Bioscience (Jena, Germany) or Trilink (San Diego, CA, USA). The PiPer pyrophosphate assay kit (Invitrogen, USA) was chosen for pyrophosphate detection. The Imprint® Methylated DNA Quantification Kit was obtained from Sigma-Aldrich (USA) for global DNA methylation assay. Rabbit anti-human DCTPP1 polycloncal antibody was obtained from Abgent (San Diego, USA Cat# NBP1-55335). The antibody against DNMT1 was purchased from Santa Cruz Biotechnology (Santa Cruz, CA, USA, Cat# sc-271729).

**Immunohistochemistry-** Breast cancer TMA slides were deparaffinized in xylene and hydrated using an ethanol gradient. Antigen retrieval was conducted by immersing slides in 10 mM citrate buffer (pH 6.0) at 120 °C for 10 min in a pressurized heating chamber. Endogenous peroxidase was blocked by incubating with freshly prepared PBS containing 3% H_2_O_2_ for 10 min. After blocking in PBS buffer containing 5% goat serum for 30 min, rabbit anti-human DCTPP1 polycloncal antibody (Abgent, USA) at dilution of 1:200 were added for incubation at 4°C overnight. Immunostaining was carried out by incubating with biotinylated goat anti-rabbit IgG (Sigma-Aldrich) and ExtrAvidin-conjugated horseradish peroxidase (Sigma-Aldrich) at dilutions of 1:800 and 1:30, respectively. Signal detection was performed using 3,3’-diamino-benzidine (DAB) as the chromogen. The slides were counterstained with hematoxylin, dehydrated, and mounted with Permount (Yiyang Instrument Inc, Shanghai, China). DCTPP1 expression were evaluated independently by two pathologists in a blinded manner using the Allred scoring system^[1]^. For each section, five fields were randomly selected. The staining was scored with 5 scales according to the percentage of DCTPP1-positive cells. For data analysis, score higher than 50 was considered high-expression.

**Construction of *DCTPP1*–knockdown MCF-7 cells-** Based on *DCTPP1* cDNA sequence, oligo siRNA candidates were designed and prepared by GenePharma (Shanghai, China). To evaluate the interfering efficacy, breast cancer cell line MCF-7 with higher expression of DCTPP1 was transfected with specific siRNA candidates using LipofectAMINE 2000 (Invitrogen) according to the manufacturer's instructions. After 48 to 72 hrs, the cells were lyzed by M-PER* Mammalian Protein Extraction Reagent (Thermo Scientific) and DCTPP1 expression level was detected by Immuoblotting assay. The siRNA oligonucleotides that efficiently inhibited DCTPP1 expression were introduced into the RNAi-Ready pSIREN-RetroQ retroviral vector (Clontech, USA) and co-transfected into 293T cells with vectors expressing the *gag* and *vsvg* genes for viral particle package according to the protocols. The viral particles was harvested and used to infect target tumor cells to generate stable *DCTPP1* knockdown cell lines. Briefly, complete DMEM medium containing viral particles was added to the target cells for 24hs’ incubation and the medium containing 1.5μg/mL puromycin (CALBIOCHEM, San Diego, CA, USA) was replaced afterward and maintained for 2 weeks. Stable transfectants (clones) were cultivated in DMEM with 10% fetal bovine serum (FBS) (Gibco) and 0.75μg/mL puromycin for at least one week before proceeding with further study. Real-time PCR and immunoblotting assays were applied to determine the efficacy of *DCTPP1* knockdown. Control vector and *DCTPP1*-shRNA-transfected MCF-7 cells were grown in RPMI-1640 medium supplemented with 10% FBS ( Gibco, Grand Island, NY, USA), 100 U/mL penicillin and 100 mg/mL streptomycin (Gibco) at 37°C in a humidified 5% CO_2_ atmosphere.

**Plasmid construction and establishment of DCTPP1 overexpressing MDA-MB-231 cells-**

The *DCTPP1* gene was amplified by PCR with the template from Hela cDNA and inserted into pIRESpuro plasmid (Invitrogen) at the EcoRI and NotI sites. MDA-MB-231 cells were transfected with 4 μg recombinant plasmid pIRESpuro-DCTPP1 or empty vector using Lipofectamine 2000 (Invitrogen) according to the manufacturer’s instructions.

48 hrs later, 1.5 μg/mL puromycin (Invitrogen) was added to the medium and maintained until the non-transfected cells died completely. Surviving puromycin-resistant cells were isolated and expanded in medium containing 0.7 μg/ml puromycin for another week. The expression of DCTPP1 in stably transfected cells was determined by Western blot and real-time PCR.

**Cell proliferation and soft agar colony assay-** The cell proliferation of stable *DCTPP1* knockdown and control MCF-7 cells was compared by the CCK-8 assay. Briefly, equal number of cells were plated into 96-well plates in sextuplicates and cultured in RPMI-1640 medium with 10% FBS. At the indicated time points, the CCK-8 reagent (Dojindo, Kumamoto, Japan) was added into the wells and incubated at 37°C for 1 hr. Measure the absorbance at 450nm using a microplate reader (Molecular Devices, Sunnyvale, CA). For soft agar colony assay, 1×10^4^ cells were suspended in 2 mL of 0.36% agar with growth medium and added in each well of a six-well plate containing a base layer of 0.72% agar. The plates were incubated at 37°C in a humidified 5% CO_2_ atmosphere for 2 weeks. Colonies from at least 5 randomly selected fields were photographed and counted for comparison.

**Cell cycle analysis-** For cell cycle analysis, 5×10^5^ of control or *DCTPP1*-deficient MCF-7 cells were seeded in six-well plates and trypsinized 24 hours later. The cell pellets were washed twice with ice-cold PBS followed by fixation with ice-cold 70% ethanol and stored at 4°C overnight. The pellets were rinsed twice with cold PBS and suspended in 500 μL PBS containing 5μL propidium iodide (PI), 0.1 mg/mL RNase A and 0.05% Triton X-100 and incubated at 37°C for 40 min in dark. The cells were acquired by FACSCalibur (BD, Stockholm, Sweden) and cell cycle was analyzed by using Cellquest software (BD).

**Mammosphere formation -** MCF-7 and MDB-MB-231 cells were cultured in DMEM/F12 (Gibco) complete medium containing B27 (Invitrogen), EGF (20 ng/ml), bFGF (10 ng/ml) (Invitrogen) and trypsin (5 ng/ml) (Gibco) at 2×10^4^/mL and incubated at 37°C with 5% CO_2_. Mammospheres were formed after 7 days and harvested for analysis.

**Cloning, expression and purification of DCTPP1 recombinant protein-**Total RNA was isolated from Hela cells and [reverse](app:ds:reverse) [transcripte](app:ds:transcription)d to cDNA. The cDNA fragment was amplified with *DCTPP1* specific primers listed in supplemental Table S1 and PCR products were digested with *Bam*H I and *Hind* III. The digested *DCTPP1* fragment was inserted into expressing plasmid pTrcHis2A containing Amp^R^ gene with fused myc and 6×His tags at the C-terminal. The recombinant plasmid was sequenced to confirm the accuracy of *DCTPP1* gene and transformed to competent E. *coli* BL21(DE3) (Tiangen Biotech, Beijing, China). The *E. coli* BL21(DE3) cells harboring pTrcHis2 A-*DCTPP1* were cultured in LB medium supplemented with 100 µg/mL ampicillin and the expression of DCTPP1-myc-6×His protein was induced in the presence of 1 mM IPTG (Sangon Biotech, Shanghai, China) for 5 hrs at 37°C. The cells were harvested by centrifugation at 4°C, resuspended in pre-cold Tris Buffer Saline (TBS) (pH7.6) supplemented with protease inhibitor cocktail Complete EDTA-free (Thermo Scientific, Hudson, USA ) and then sonicated. Cell lysate was centrifuged at 15 000 ×g for 15 min to remove cell debris and unbroken cells. The supernatant was loaded on a Ni-NTA agarose column (Qiagen, Hilden, Germany). The column was washed with TBS containing [gradient](app:ds:gradient) concentration of imidazole, and DCTPP1-myc-6×His protein was finally eluted with TBS containing 0.3 M imidazole. Fractions containing DCTPP1 fusion protein were pooled and condensed by 3kDa Centrifugal Filter Devices (Millipore, Massachusetts, USA). Protein concentration was measured with BCA Protein Assay Reagent Kit (Thermo Scientific).

**Homolog modeling of human DCTPP1 structures-**The X-ray crystal structure of the tetrameric mouse dCTPase 1 (Protein Data Bank entry 2OIG, resolution 3.3 Å)^[2]^ was used as the template to construct the human monomeric, dimeric and tetrameric DCTPP1 structure models. The sequence of the human DCTPP1 was retrieved from UniProt (http://www.uniprot.org/uniprot/Q9H773). According to the secondary structure information of the template, the sequence alignments were adjusted manually to be more comparable. The 3D models of human DCTPP1 and its 5-methyl-dCTP-bound tetramer were generated using MODELLER software (version 9.12)(Sali and Blundell 1993)^[3]^.

**Docking-**Grid-based ligand docking from energetics (GLIDE) software^[4]^ (Schrödinger suite 2009) was used for predicting dCTP and dGTP binding poses based on the 5-methyle-dCTP-bound tetramer structure model. To validate the docking approach, the 5-methyl-dCTP, which bound into the catalytic site of DCTPP1, was extracted from the modelled complex structure and used to perform docking.

The simulated 5-methyl-dCTP-bound structure was prepared using the ‘Protein Preparation Wizard’ implemented in Schrödinger suite. Hydrogen atoms and charges were added during a brief relaxation performed with the ‘Interactive Optimizer’ option and a restrained partial minimization was terminated when the root-mean-square deviation (RMSD) reached a maximum value of 0.3 Å in order to relieve steric clashes. Amino acid residues located within 15 Å from the centroid of bound 5-methyl-dCTP were defined as the binding pocket for docking simulations. Three-dimensional structures of 5-methyl-dCTP, dCTP and dGTP were prepared with ‘LigPrep’ tool of Schrödinger suite.

In the docking process, the dCTP and dGTP were docked into the binding site using GLIDE 5.5 with extra-precision (XP) to generate the poses. The final binding models were selected based on the Glide scoring function (G-Score) as well as visual inspection.

**Catalytic activity assay of DCTPP1-** The substrates of DCTPP1 tested in this study contains canonical and non-canonical (d)NTPs. The reactions were conducted in a 60µl reaction mixture of 100 mM Tris-HCl buffer (pH 7.5) containing 1 mM MgCl_2_, 100µM substrates and 1.2 µg purified DCTPP1 protein. Reaction mixtures without enzyme or substrates were carried out as background controls. The reaction mixtures were incubated at 37°C for 10min and terminated at 72°C for 10 min. The PiPer pyrophosphotate assay was conducted according to the manufacturer's instructions (Invitrogen). Briefly, 50 µl of the reaction mixtures or standard PPi was mixed with 50 µL working solution, incubated for 1 hr at 37°C protected from light and detected the absorbance at 565 nm. The corresponding PPi concentration was calculated according to the standard curve. The results were analyzed by GraphPad Prism 5.0 (Graphpad Software. San Diego,USA).

**Real-time PCR-**Total RNA was extracted using Trizol reagent (Invitrogen) according to the manufacturer’s instructions. 2 μg of total RNA was reverse transcripted by random primer using RevertAid First Strand cDNA Synthesis Kit (Thermo Scientific). Quantitative real-time PCR was performed by using commercial kits (TaKaRa). Primers used in this study were listed in supplementary table 1. *GAPDH* was applied as the internal control. Gene expression was indicated by 2^-△△CT^ using NC-shRNA transfected MCF-7 cells or mammospheres as control samples.

**Measurement of dCTP and 5-methyl-dCTP content by using liquid chromatography tandem mass spectrometry (LC-MS/MS)**

Preparation of calibration standards

dCTP^13^C,^15^N was used as the internal standard for dCTP and 5-methyl-dCTP. The internal standard stock solutions as well as dCTP and 5-methyl-dCTP stock solutions were prepared separately by dissolving the analytes in LC–MS grade water at a concentration of 1 mg/mL. All solutions were subsequently frozen at −70 °C. All further standard and internal standard samples were obtained by diluting the stock solutions with methanol.

LC-MS/MS

Liquid chromatography was performed using an Agilent 1200 HPLC system (Agilent Technologies, CA, USA), and separation was carried out at 40°C using a ZIC-HILIC column (2.1 mm × 100 mm, 3 μm; Merck Sequant, Umea, Sweden). Isocratic elution was performed. The mobile phase consisted of methanol and water (75:25, v/v, the aqueous phase contained 20 mM ammonium formate and was adjusted to pH 3 with formic acid). The flow rate was set at 300 μL/min, and the injection volume was 5 μL. The HPLC system was coupled to an Agilent 6410 triple quadrupole mass spectrometer (Agilent Technologies, CA, USA). The eluent from the first 1 min of the run was diverted to the waste to avoid potential contamination and ion suppression in the ion source. An ESI in positive ionization mode was used. The ESI source parameters were set as follows: nebulizer gas (N2) flow rate, 10 L/min; gas temperature, 350°C; capillary voltage, 3000 V; and nebulizer pressure, 45 psi. The mode of multiple reaction monitoring (MRM) was used to identify and quantify dCTP (transition: m/z 468.1 [M + H]+→112.1, fragmentor 120 V, collision energy 20 eV) , 5mdCTP (transition: m/z 482.1 [M + H]+→126.1, fragmentor 140 V, collision energy 20 eV), and the internal standard dCTP13C,15N (transition: m/z 480.1 [M + H]+→119.1, fragmentor 125 V, collision energy 20 eV). The analytic data were processed using the MassHunter software package (Agilent Technologies, CA, USA), which contained qualitative and quantitative analysis modules.

Sample preparation procedure

One hundred microliters of sample were mixed with 50 μL of methanol, containing the internal standard at a concentration of 20 ng/mL (dCTP^13^C,^15^N). Five microliters of this solution were injected into the LC-MS/MS.

**Author contributions**

SFF and XLL performed most of the experiments. JP performed the cell culture and mammosphere formation experiment. WJQ performed immunohistochemistry. TYB and ZL performed the LC-MS analysis. HZM and ZJ performed the computational simulation. SFF and WY wrote the manuscript. ZGP and GHL initiated the projects and revised the manuscript. ZY and WY designed the projects and revised the manuscript. All authors have read the manuscript.

Table S1 Oligonucleotides used in the study:

| **Name** | **Sequence** | **Purpose** |
| --- | --- | --- |
| *DCTPP1*-F Primer | 5'-CCCGGATCCATGTCTGTGGCCGG-3' | cDNA amplication |
| *DCTPP1*-R Primer | 5'-CCCAAGCTTCTAGGTTGAGGTCTG-3' |
| *DCTPP1*-F Primer | 5'-CGCCTCCATGCTGAGTTTG-3' | Real-time PCR |
| *DCTPP1*-R Primer | 5'-CCAGGTTCCCCATCGGTTTTC-3' |
| *GAPDH*-F Primer | 5'-AAGGTGAAGGTCGGAGTCAAC-3' | Real-time PCR |
| *GAPDH*-R Primer | 5'-GGGGTCATTGATGGCAACAATA-3' |
| *DNMT1-*F Primer | AGGCGGCTCAAAGATTTGGAA | Real-time PCR |
| *DNMT1-R Primer* | GCAGAAATTCGTGCAAGAGATTC |
| ShRNA1 sense | 5'-gatccGCCCTTCAAGAGGAGCTTATTCAAGAGATAAGCTCCTCTTGAAGGGCTTTTTTACGCGTg-3' | *DCTPP1* Knockdown |
| ShRNA1 antisense | 5'-aattcACGCGTAAAAAAGCCCTTCAGAGGAGCTTATCTCTTGAAGCTCCTCTTGAAGGGCg-3' |
| ShRNA2 sense | 5'-gatccGCCGCAAGTATACAGAATTGTTCAAGAGACAATTCTGTATACTTGCGGTTTTTTACGCGTg-3' |
| ShRNA2 antisense | 5'-aattcACGCGTAAAAAACCGCAAGTATACAGAATTGTCTCTTGAACAATTCTGTATACTTGCGGCg-3’ |
| NC-shRNA sense | 5'-gatccGTGCGTTGCTAGTACCAACTTCAAGAGATTTTTTACGCGTg -3' | Negative Control |
| NC-shRNA antisense | 5'-aattcACGCGTAAAAAATCTCTTGAAGTTGGTACTAGCAACGCACg -3' |

**Supplemental figure 1. Detection of DCTPP1 expression in multiple cancer cell lines by Western blotting**

**Supplemental figure 2. Optimization of DCTPP1 enzymatic activity** (A) Recombinant DCTPP1 was expressed in *E.coli.* and purified as described in “supplementary materials”. The purity of DCTPP1 was analyzed by 12% SDS-PAGE. The deduced molecular weight of recombinant DCTPP1 with 6×His and Myc tag was 21.7kD. (B) Comparison of 4 divalent cations as co-factors during catalytic reaction. 500 μM dCTP was conducted as substrate under 1 mM of different cations. The reaction mixture without substrates was used as a background control. (C) Mg^2+^-dependent enzymatic activity of DCTPP1. The reaction was performed under increased Mg^2+^ concentration. No activity was detected without the addition of metals. The maximum activity reached in the reactions containing 10 mM Mg^2+^. (D) pH optimization for DCTPP1 activity. The activity under different pH was normalized to the activity of pH 8.7. Each point and error bar represented the mean ± SD of at least three independent reactions.

**Supplemental figure 3. Comparison of DNMT1 expression.** The mRNA level of DNMT1 in MCF-7 with DCTPP1 knockdown (A) and MDA-MB-231 with DCTPP1 overexpression (B) was measured by Real-time PCR. Western blot analysis was also performed to examine the protein level of DNMT1 after DCTPP1 knock down (C) and overexpression (D).

Reference

[1] Harvey JM, Clark GM, Osborne CK, Allred DC. Estrogen receptor status by immunohistochemistry is superior to the ligand-binding assay for predicting response to adjuvant endocrine therapy in breast cancer. J Clin Oncol. 1999; 17(5):1474-81.

[2] Wu B, Liu Y, Zhao Q, Liao S, Zhang J, Bartlam M, et al. Crystal structure of RS21-C6, involved in nucleoside triphosphate pyrophosphohydrolysis. J Mol Biol. 2007; 367(5):1405-12.

[3] Martí-renom MA, Stuart AC, Fiser A, Sánchez R, Melo F, Šali A. Comparative protein structure modeling of genes and genomes. Annu Rev Biophys Biomol Struc. 2000; 29(1):291-325.

[4] Friesner RA, Banks JL, Murphy RB, Halgren TA, Klicic JJ, Mainz DT, et al. Glide: a new approach for rapid, accurate docking and scoring. 1. Method and assessment of docking accuracy. J Med Chem. 2004; 47(7):1739-49.
